# Extensive Uterine Necrosis and Peritonitis Following Clandestine Abortion Attempt: A Case Report

**DOI:** 10.1002/ccr3.71689

**Published:** 2025-12-15

**Authors:** Hamidou Soumana Diaouga, Yahouza Boka Tounga, Adama Saidou, Kadi Ide, Madi Nayama, Sani Rachid

**Affiliations:** ^1^ Obstetrics and Gynecology Service, Maternity Issaka Gazobi Abdou Moumouni University Niamey Niger; ^2^ Department of Digestive Surgery, National Hospital of Niamey Abdou Moumouni University Niamey Niger; ^3^ Department of Digestive Surgery, General Referral Hospital of Niamey Abdou Moumouni University Niamey Niger

**Keywords:** case report, obstetrical hysterectomy, peritonitis, unsafe abortion, uterine perforation

## Abstract

Clandestine abortion remains a prevalent health concern in developing countries, often resulting in severe complications, including uterine secrosis with generalized peritonitis necessitating life‐saving hysterectomy, which results in permanent infertility. A 18‐year‐old primigravida patient presented to our department with severe abdominal pain and vaginal bleeding following a clandestine abortion attempt, 15 days prior. Furthermore, the patient exhibited symptoms of uncontrollable vomiting and diarrhea that began 4 days prior to admission. Upon further examination, she was diagnosed with peritonitis. We performed an emergency laparotomy. It was discovered that the uterus was necrotic and that the foetus was in the abdominal cavity with its placenta. A hysterectomy was necessary to save the patient's life, resulting in permanent infertility. The patient's postoperative course was uncomplicated, and she was discharged from hospital on the 12th day after surgery. This case highlights the need for political and medico‐social actions facilitating access to sexual and reproductive education and to voluntary termination of pregnancy in a legal and medicalized context in our regions.

## Key Clinical Massage

In developing countries, clandestine abortion remains a prevalent issue, often resulting in misdiagnosis, severe uterine necrosis and generalized peritonitis. This can necessitate hysterectomy with permanent infertility. It is important for practitioners to be able to identify this situation when it arises, even when the serum β‐human chorionic gonadotropin level is normal.

## 1 Introduction

An unsafe abortion is defined as a procedure for terminating an unintended pregnancy carried out either by a person lacking the necessary skills or in an environment that does not conform to minimal medical standards or both [1]. Illegal abortion remains a significant public health concern in Niger and other developing countries, where it is prevalent but often underestimated due to its illegal and clandestine nature [1, 2]. Worldwide, 73 million induced abortions are carried out each year, 45% of which are unsafe. 97% of these abortions take place in underdeveloped countries in sub‐Saharan Africa and central and south Asia [3, 4]. It is responsible for significant maternal morbidity and mortality. For instance, in Africa, unsafe abortions accounted for approximately one‐third of all maternal mortalities [3, 5].

We hereby present a case of uterine necrosis following clandestine abortion, which was discovered incidentally during a laparotomy in an 18‐year‐old patient treated in our department for acute peritonitis. The significance of this extreme case lies in emphasizing the necessity for political and medico‐social actions to facilitate access to sexual and reproductive education, as well as to voluntary termination of pregnancy in a legal and medicalised context within our regions.

## 2 Case History/Examination

A 18‐year‐old black African woman (gravida 1, para 0) presented to our emergency department with diarrhea, uncontrollable vomiting and abdominal pain 2 weeks following a clandestine abortion attempt after an unwanted 14 weeks pregnancy that occurred outside the context of marriage. Apart from the recent clandestine abortion attempt, she has no significant medical or surgical history. The patient revealed during the interview that she had undergone a clandestine abortion 2 weeks prior. The procedure was discreetly conducted at home by the patient herself. The procedure involved the insertion of a millet stalk into the uterine cavity through the vagina, with the objective of inducing an interruption of the pregnancy. Following the procedure, the patient reported a history of pelvic pain and vaginal bleeding. From day 2 to day 13 following the procedure, she reported ongoing bleeding, abdominal pain and fever. The patient did not inform her family and had not consulted a health professional.

From day 14 to day 17 after the procedure, she complained of persistence of previous signs associated with uncontrollable vomiting and diarrhea. The patient's family was aware of her health problems, but she kept her pregnancy and attempted abortion a secret. She had previously attempted self‐medication with analgesics and antipyretics, but to no improvement. Due to the exacerbation of symptoms, the patient was presented to our department by her family.

Upon presentation on the 18th day after the clandestine abortion attempt procedure, the patient was found to have hypotension (blood pressure: 90/60 mmHg), tachycardia (heart rate: 128 bpm), fever (temperature: 40.1°), and pallor. The physical examination of the abdomen revealed that it was distended with generalized abdominal guarding, which is indicative of acute generalized peritonitis. Upon pelvic examination, there was evidence of vaginal trauma. It was established that the cervix had trauma. The palpation of the uterus was challenging due to the pain. The rectal examination was painful and revealed the presence of fluid diarrhea. The Douglas's wall had become distended.

### 2.1 Differential Diagnosis

This clinical symptomatology allows us to consider differential diagnoses such as uterine perforation, bowel perforation following a bacterial infection, acute appendicitis, or cholecystitis.

### 2.2 Investigations and Treatment

Laboratory investigations showed a white blood cell count of 26.35. 10^3^/mm^3^ (4–10. 10^3^/mm^3^), hemoglobin level of 7.4 g/dL (11–16 g/dL), platelets count of 248. 10^3^/mm^3^ (100–350. 10^3^/mm^3^), the C‐reactif protein at 116 mg/L (< 5 mg/L). The serum β‐human chorionic gonadotropin (β‐HCG) level was < 5 UI/L. Liver and renal panel were normal.

A plain abdominal X‐ray was performed, revealing diffuse grayness without pneumoperitoneum. This suggests a diagnosis of acute generalized peritonitis. Unfortunately, the CT scan could not be performed as it was not available. The diagnosis of acute generalized peritonitis was confirmed. The patient was given a transfusion of two units of whole blood.

A resuscitation protocol was established based on: hydro‐electrolytic rehydration (alternating saline serum: 500 mL, ringer serum: 500 mL and glucose serum: 500 mL), antibiotic therapy (ceftriaxone: 2 g/day for 5 days and metronidazole 500 mg/8 h for 5 days), analgesic (paracetamol: 1 g/8 h).

**FIGURE 1:**
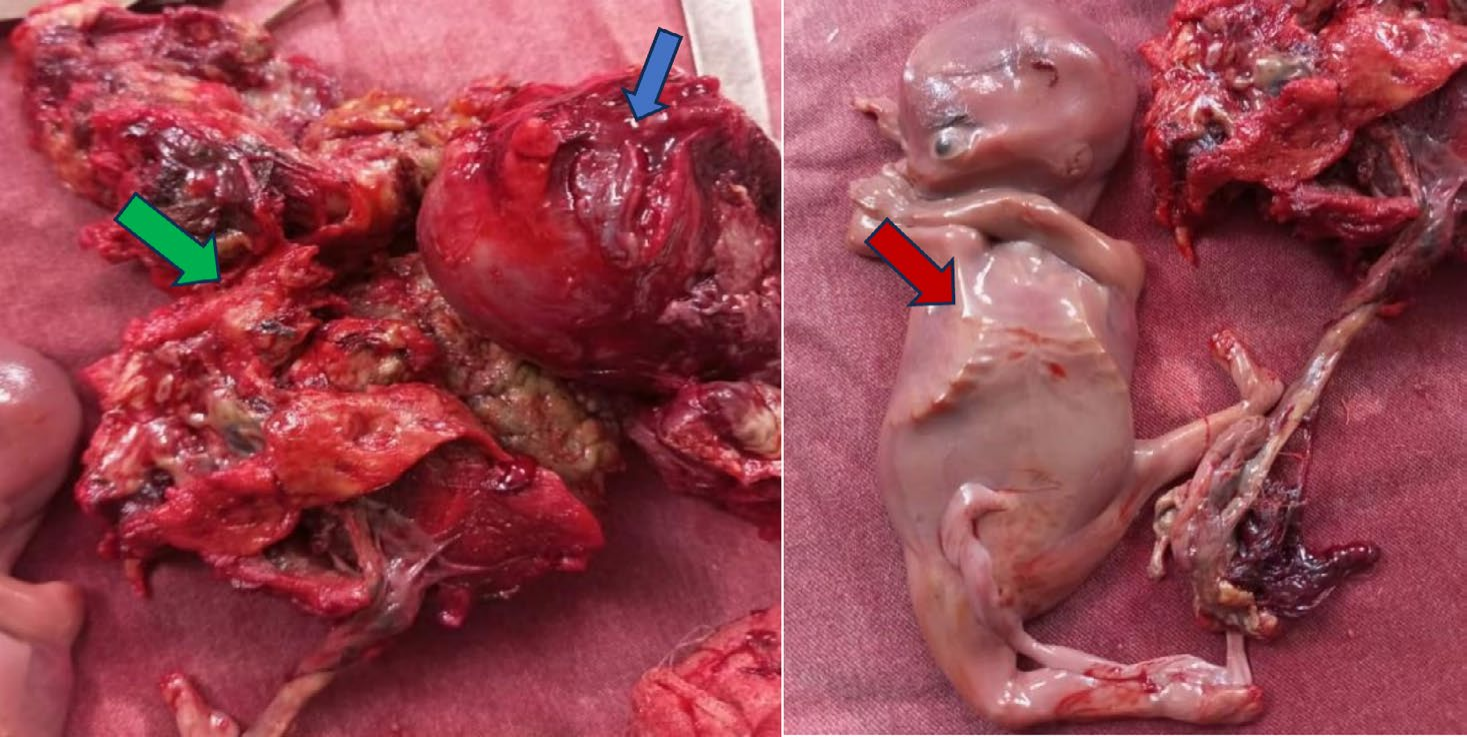
Post‐operative image: Note a perforated and necrotic uterus (blue arrow), a macerated placenta (green arrow) and a macerated fetus corresponding to a gestational age of 14 weeks (red arrow).

In view of the symptoms indicating peritonitis, an exploratory laparotomy was performed. Intraoperatively, 1500 cc of purulent fluid was aspirated but not sent for bacterial cultures because this was not an available service in our setting. During the exploration, the presence of diffuse false membranes was identified. It was evident that there was perforation and extensive necrosis of the uterine fundus. A macerated foetus, corresponding to 14 weeks of pregnancy, was found in the abdominal cavity with its placenta, part of which had remained adherent to the uterine cavity. Following meticulous adhesiolysis and removal of the foetus and placenta, a total hysterectomy was performed (Figure 1). Following confirmation of adequate hemostasis, the abdominal cavity was irrigated with isotonic saline and the abdominal wall was closed in layers, with two drainage tubes inserted.

## 3 Conclusion and Results

The patient's postoperative course was favorable, and she was discharged from hospital in good health on post operative day 12. She was satisfied with the treatment received. Upon discharge, the patient was informed about her future obstetric concerns. After a period of 1 month, the patient has not reported any complaints, and a physical examination has not revealed any complications. The patient is currently under the surveillance of a psychiatrist and the surgical team.

In conclusion, complications arising from clandestine abortion attempts can lead to uterine necrosis, which can require a life‐saving hysterectomy. It is imperative that this situation is identified at the outset of the presentation. In order to prevent clandestine abortion and its associated complications, it is essential that social and political actions are taken, alongside the provision of comprehensive sexual education and access to safe medical care.

The Table 1 present the patient timeline.

**TABLE 1:** Patient timeline.

Date	Events	Diagnosis and actions taken
August 10, 2024	The patient reports a 4‐month pregnancy that was not desired In Niger, it is widely considered unacceptable for a woman to become pregnant outside of marriage, and this is viewed as a shameful and dishonorable act for both the woman and her family The patient wishes to terminate the pregnancy, but it should be noted that abortion is illegal and punishable by law in Niger	The patient has made a clandestine abortion attempt at home, using a millet stalk inserted into the vagin to push into the uterine cavity
August 11, 2024	Vaginal bleeding persists and moderate pelvic pain develops	The patient has chosen not to disclose her story and has not requested medical consultation
From August 12, to August 24, 2024	Fever There is a continued occurrence of vaginal bleeding of a dark, foul‐smelling nature The patient is experiencing exacerbated pelvic pain that has now spread to the rest of the abdomen	The family becomes aware of the situation, but the patient kept her pregnancy and the attempted abortion a secret Self‐medication involving the use of analgesics and antipyretics
From August 24 to August 27, 2024	Diarrhea and a foul‐smelling vaginal discharge Fever, abdominal pain and uncontrollable vomiting	Self‐medication with drugs whose nature has not been specified
August 28, 2024	The patient was admitted to our emergency department She presented with acute generalized peritonitis, fever, diarrhea and a foul‐smelling vaginal discharge	Antibiotic therapy, analgesia, antipyretics Blood transfusion Rehydratation (1.5 L of saline, Ringer's solution and, glucose) We performed an exploratory laparotomy, during which we made the intraoperative diagnosis of extensive uterine necrosis and a foetus in the abdominal cavity. We then performed a total hysterectomy
September 7, 2024	The postoperative course was uncomplicated	The patient was discharged on the twelfth day after surgery

## 4 Discussion

This report outlines a case of extensive uterine necrosis and acute generalized peritonitis following a clandestine abortion attempt in an 18‐ year‐old primigravida who had been managed in a limited‐resource setting. We then compared the diagnostic, therapeutic, and prognostic aspects of this case report with those reported in the literature.

This case report is distinct from others in the literature in terms of the severity of the clinical presentation (perforation and extensive necrosis of the uterus, and expulsion of the foetus into the abdominal cavity) and biological findings (serum β‐human chorionic gonadotropin level was < 5 UI/L), which constituted a diagnostic pitfall in this context where the patient stated that she had never been pregnant.

In this case report, the patient's symptoms of diarrhea and peritonitis were erroneously attributed to a bowel perforation following bacterial infection or acute appendicitis.

The main causes of clandestine abortion are socio‐cultural considerations that stigmatize women who become pregnant outside of marriage, the criminalisation of abortion, and young girls' lack of access to reproductive health services.

In this case report, the patient was not married and the pregnancy was both spontaneous and unwanted. The patient had not received a formal education and had no knowledge of reproductive health. She resided in a rural setting and was not in employment. It is important to note that, in Niger and in most African countries, voluntary termination of pregnancy remains illegal, whether medicalized or not [1, 2, 3, 6].

Although clandestine abortion is a common occurrence in developing countries, there have been no documented cases of extreme situations, such as extensive necrosis of the uterus, followed by expulsion of the foetus into the abdominal cavity.

At the level of the presentation, symptoms in this case report included persistent vaginal bleeding, fever and abdominal pain. As outlined in the literature, these signs have been documented in the majority of reported cases [1, 2, 3, 6, 7, 8].

Despite this specific clinical presentation, the social stigma associated with pregnancy in unmarried women, which is considered a dishonor for the woman and her family, and the fear of criminal prosecution following a clandestine abortion attempt were key factors contributing to delayed diagnosis.

In this case report, complications manifested immediately after the clandestine abortion procedure, as evidenced by symptoms including excessive vaginal bleeding and severe abdominal pain. This finding aligns with the majority of cases documented in the literature [1, 3, 5, 7]. The diagnosis was made incidentally during the surgical procedure for acute generalized peritonitis. Similar cases of accidental intraoperative diagnosis have been documented in the literature [1, 2, 3]. In the present case report, the time between the clandestine abortion procedure and the occurrence of complications requiring the patient to consult was 17 days. Early consultation by the patient would have facilitated earlier diagnosis allowing conservative management. According to the literature on the subject, the average time between the clandestine abortion procedure and the occurrence of complications requiring the patient to consult is ten days, with extremes of 4–40 days [1, 3, 5, 7].

From a pragmatic perspective, the preliminary step in the diagnostic process is to consider the possibility of an unsafe abortion in cases of non‐specific signs in a woman of childbearing age. The second step in the process is to conduct a detailed interview with the patient and carry out the relevant biochemical and radiological examinations. Serum β‐human chorionic gonadotropin dosage is typically the primary investigative method, allowing confirmation of the presence of a pregnancy. However, the information gathered is often limited because the serum β‐hCG is frequently undetectable a few days after abortion or termination of pregnancy, as was the case in this case report.

Ultrasonography of the abdomen and pelvis is typically the primary investigative imaging method. Ultrasound is particularly useful in underdeveloped countries due to the following reasons: it is readily available, affordable, does not involve ionizing radiation, and can be performed at the bedside or in the operating room. However, this should not preclude computed tomography (CT), magnetic resonance imaging (MRI), or radiographic evaluation. Abdominal radiography is used to demonstrate air‐fluid levels [6, 7, 9].

In terms of the management of clandestine abortion complications, the literature has documented two therapeutic approaches: conservative treatment and surgical treatment (laparotomy or endoscopic approach). The selection of a therapeutic method is determined by the type and severity of complications.

In this case report, the patient was in peritonitis and had extensive uterine necrosis. Conservative treatment was not a viable option for our patient. We performed a laparotomy, followed by a life‐saving hysterectomy. This approach has been employed by several authors in similar cases [2, 4].

Most authors have reported conservative management in cases of uterine perforation, consisting of reviving the edges of the perforation, followed by its closure with interrupted stitches [1, 2, 3, 7, 9].

With regard to the prognosis, clandestine abortion is associated with significant morbidity and mortality. The stage of pregnancy, the qualifications of the operator (i.e., experienced health professional, the patient herself or an operator without medical knowledge), and the conditions or the nature of instrument used to induce the abortion are the determining factors of complications from clandestine abortions.

In this particular case report, the patient had introduced a millet stalk into the uterine cavity. This resulted in uterine perforation, followed by significant uterine necrosis and expulsion of the foetus into the abdominal cavity. As outlined in the literature, there have been several documented cases involving various traumatic instruments [1, 2, 3, 6, 7]. For instance, the endo‐uterine maneuvers described in the study by Ka et al. [2] and Aule et al. [8] were performed using wooden rods and metal probes, respectively. As outlined in two case reports [2, 4], a hysterectomy was performed to save the patient's life, resulting in definitive sterility. This is also the case with our patient. Notably, despite her being a primigravida, we successfully performed a total hysterectomy.

### 4.1 Take‐Home Messages for Clinicians in Low‐Resource Settings

- An attempted clandestine abortion can lead to extensive uterine necrosis, followed by expulsion of the foetus into the abdominal cavity. This can mimic an abdominal pregnancy or a pelvic abscess.
- It is not uncommon for patients to claim that they have never been pregnant, and in such cases the serum β‐hCG test may be negative due to the delay in seeking medical advice. This can present a diagnostic pitfall.

## Author Contributions

**Hamidou Soumana Diaouga:** data curation, formal analysis, investigation, methodology, writing – original draft, writing – review and editing. **Yahouza Boka Tounga:** conceptualization, data curation, methodology, writing – original draft. **Adama Saidou:** methodology, visualization, writing – review and editing. **Kadi Ide:** formal analysis, methodology, validation, visualization, writing – review and editing. **Madi Nayama:** supervision, validation, writing – review and editing. **Sani Rachid:** project administration, supervision, validation, writing – review and editing.

## Funding

The authors have nothing to report.

## Ethics Statement

The case report was conducted following ethical standards and patient confidentiality was maintained.

## Consent

Written informed consent was obtained from the patient for publication of this case report and any accompanying images. A copy of the written consent is available for review by the Editor‐in‐Chief of this journal.

## Conflicts of Interest

The authors declare no conflicts of interest.

## Data Availability

The data used to support the findings of this study are available from the corresponding author upon reasonable request.
